# Impact of racial disparities in follow-up and quality of colonoscopy on colorectal cancer outcomes

**DOI:** 10.1093/jnci/djae140

**Published:** 2024-07-24

**Authors:** Oguzhan Alagoz, Folasade P May, Chyke A Doubeni, A Mark Fendrick, Vahab Vahdat, Chris Estes, Travelle Ellis, Paul J Limburg, Durado Brooks

**Affiliations:** Department of Industrial & Systems Engineering, University of Wisconsin-Madison, Madison, WI, USA; Department of Medicine, University of California Los Angeles (UCLA) Health and UCLA Kaiser Permanente Center for Health Equity, Los Angeles, CA, USA; Department of Family and Community Medicine, College of Medicine, Comprehensive Cancer Center, Wexner Medical Center, The Ohio State University, Columbus, OH, USA; Department of Internal Medicine and Department of Health Management and Policy, Division of General Medicine, University of Michigan, Ann Arbor, MI, USA; Institute for Healthcare Policy and Innovation, University of Michigan, Ann Arbor, MI, USA; Exact Sciences Corporation, Madison, WI, USA; Exact Sciences Corporation, Madison, WI, USA; Exact Sciences Corporation, Madison, WI, USA; Exact Sciences Corporation, Madison, WI, USA; Exact Sciences Corporation, Madison, WI, USA

## Abstract

**Background:**

The benefits of colorectal cancer (CRC) screening programs rely on completing follow-up colonoscopy when a noncolonoscopy test is abnormal and on quality of colonoscopy screening as measured by the endoscopists’ adenoma detection rate. Existing data demonstrate substantially lower follow-up colonoscopy rates and adenoma detection rate for Black Americans than White Americans. However, the contributions of racial differences in follow-up colonoscopy and adenoma detection rate on CRC outcomes have not been rigorously evaluated.

**Methods:**

We used established and validated CRC-Adenoma Incidence and Mortality (CRC-AIM) model as our analysis platform, with inputs from published literature that report lower follow-up colonoscopy rates and adenoma detection rate in Black adults compared with White adults (15% and 10% lower, respectively). We simulated screening with annual fecal immunochemical test, triennial multitarget stool DNA, and colonoscopy every 10 years between ages 45 and 75 years using real-world utilization of the screening modalities vs no screening. We reported lifetime outcomes per 1000 Black adults.

**Results:**

Elimination of Black-White disparities in follow-up colonoscopy rates would reduce CRC incidence and mortality by 5.2% and 9.3%, respectively, and improve life-years gained with screening by 3.4%. Elimination of Black-White disparities in endoscopists’ adenoma detection rate would reduce CRC incidence and mortality by 9.4% and improve life-years gained by 3.7%. Elimination of both disparities would reduce CRC incidence and mortality by 14.6% and 18.7%, respectively, and improve life-years gained by 7.1%.

**Conclusions:**

This modeling study predicts eliminating racial differences in follow-up colonoscopy rates, and quality of screening colonoscopy would substantially reduce Black-White disparities in CRC incidence and mortality.

In the United States, colorectal cancer (CRC) incidence and mortality rates have decreased over time, but racial and ethnic disparities persist (1). Compared with White Americans, Black Americans experience higher CRC incidence, higher rates of advanced stage at presentation, and lower survival rates (2,3). In 2019, the age-adjusted CRC incidence and mortality rates were 118 and 54 per 100 000 Black adults aged 50 years and older compared with 96 and 41 per 100 000 White adults, translating to approximately 23% and 31% higher risk of CRC incidence and mortality among Black adults, respectively (1).

Adherence to screening has historically been identified as the main driver of racial disparities in CRC incidence and mortality. A study using data from the National Health Interview Survey (NHIS) for 2005 showed a substantially higher proportion of US adults aged 50-75 years who were up to date with screening among White (52%) than Black people (39%) (4). The gap has narrowed in recent years. In a study using 2019 NHIS data, a similar proportion of Black (69.5%) and White (69.8%) adults aged 50-75 years were up to date with CRC screening (5). Persistent Black-White disparities in CRC incidence, mortality, and survival despite similar screening participation suggest that other factors may be the primary contributors for these disparities (6).

Colonoscopy and stool-based testing such as the fecal immunochemical test and multitarget stool DNA test are the most commonly used CRC screening tests in the United States. The effectiveness of screening with stool-based tests in reducing the risk of CRC deaths relies on completing follow-up colonoscopy after an abnormal test result, while the effectiveness of colonoscopy for primary screening varies with the quality of the procedure, as measured by the adenoma detection rate (7,8).

Existing data demonstrate that follow-up colonoscopy rates for abnormal stool-based tests are substantially lower for Black Americans than their White counterparts (9-16). Delays in follow-up colonoscopy increase the risk for CRC incidence, advanced stage at presentation, and mortality (17-19). Similarly, a higher proportion of Black Americans than White Americans receive screening colonoscopies from endoscopists with a lower adenoma detection rate, defined as the proportion of individuals undergoing a complete colonoscopy who had at least 1 adenoma detected (20-24). Adenoma detection rate is considered the most important indicator of quality in colonoscopy, and higher adenoma detection rate is associated with better patient outcomes, including reduced risk of interval CRC (CRC diagnosed between screening colonoscopies) (24).

Despite evidence of the potential for differences in follow-up colonoscopy and adenoma detection rate to contribute to racial disparities in CRC outcomes, their impact on CRC incidence and mortality, and life-years gained with screening, remains understudied. We used an established and validated CRC simulation model to estimate the impact of these disparities on CRC health outcomes.

## Methods

The study is exempt from human subjects’ research because of the use of published and publicly available aggregate data. We used the CRC–Adenoma Incidence and Mortality (CRC-AIM) microsimulation model that was previously calibrated and validated to CRC incidence and mortality in the United States (25). CRC-AIM is briefly summarized below, and the details are included in a previous publication (25).

### Model description

**Figure 1.**
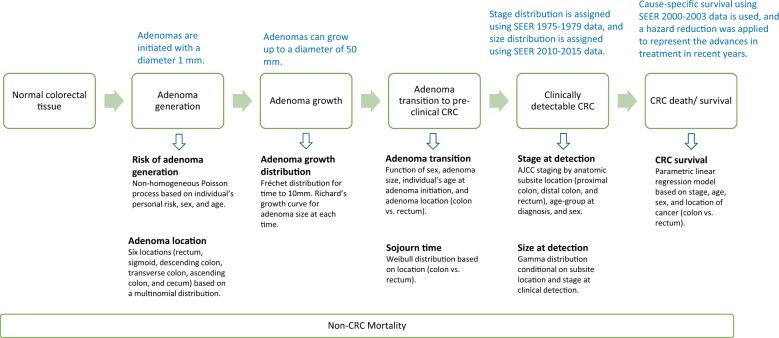
Overview of Colorectal Cancer–Adenoma Incidence and Mortality microsimulation model [adapted from Vahdat et al. (25)]. AJCC = American Joint Committee on Cancer; CRC = colorectal cancer; SEER = Surveillance, Epidemiology, and End Results Database.

CRC-AIM simulates CRC-related events for the average-risk US adults by modeling the CRC natural history using 5 interrelated subcomponents (Figure 1): 1) adenoma generation that assigns a risk of developing adenomas based on location and an individual’s sex, age, and baseline risk; 2) adenoma growth based on tumor location (colon and rectum); 3) transition from adenoma to preclinical cancer based on tumor location, sex, size, and age at initiation of adenoma; 4) transition from preclinical cancer to clinically detectable cancer based on tumor location; and 5) survival after diagnosis based on cancer stage, age, sex, and location using National Cancer Institute’s Surveillance, Epidemiology, and End Results database. Key parameters of CRC-AIM were informed by high-quality data sources (see Table 1). CRC-AIM was validated against the United Kingdom Flexible Sigmoidoscopy Screening Trial and National Cancer Institute’s 3 Cancer Intervention and Surveillance Modeling Network CRC models (25).

**Table 1.** List of data sources used by CRC–Adenoma Incidence and Mortality^a^

Name	Description	Source
Population demographics and other-cause mortality		
Population demographic characteristics	Used directly in the model	US census data (41)
All-cause mortality	Used directly in the model	2017 US life tables (42)
Natural history		
Risk of adenoma, adenoma growth, and transition to preclinical cancer	Calibrated against published literature	Literature (43-47)
Adenoma location	Six locations (rectum, sigmoid, descending, transverse, ascending, cecum) are assigned based on 9 autopsy studies.	Literature (48-57)
Stage distribution of clinically detected CRCs	Used SEER 1975-1979 data by age, sex, and location (colon and rectum)	SEER (1)
Average size of clinically detected CRCs	Calibrated against SEER 2010-2015 data for individuals younger than 50 years	SEER (1)
CRC incidence	Calibration target from SEER 1975-1979 data was used	SEER (1)
Screening		
Sensitivity of colonoscopy	Directly used (adenomas <6 mm: 0.75; adenomas 6-9 mm: 0.85; adenomas >9 mm: 0.95; preclinical CRC: 0.95)	Modeling study for the USPSTF 2021 CRC screening guideline update (26) and literature (58)
Sensitivity of fecal immunochemical test	Calibrated against the values provided by the modeling study for the USPSTF 2021 CRC screening guideline update (adenomas <6 mm: 0.03; adenomas 6-9 mm: 0.14; adenomas >9 mm: 0.22; preclinical CRC: 0.74)	Modeling study for the USPSTF 2021 CRC screening guideline update (26,27)
Sensitivity of multitarget stool DNA	Calibrated against the values provided by the modeling study for the USPSTF 2021 CRC screening guideline update (adenomas <6 mm: 0.09; adenomas 6-9 mm: 0.33; adenomas >9 mm: 0.42; preclinical CRC: 0.94)	Modeling study for the USPSTF 2021 CRC screening guideline update (26,27)
Specificity of screening tests	0.86 for colonoscopy; 0.97 for fecal immunochemical test; 0.81 for multitarget stool DNA	Modeling study for the USPSTF 2021 CRC screening guideline update (26,27) and literature (59)
Reach of colonoscopy	The reach of colonoscopy linearly decreases from rectum (100%) to cecum (95%).	Modeling study for the USPSTF 2021 CRC screening guideline update (26)
Complications associated with colonoscopy	Age-specific risks of serious gastrointestinal events, other gastrointestinal events, and cardiovascular events associated with colonoscopy are included.	Modeling study for the USPSTF 2021 CRC screening guideline update (26)
Management of false-positive test results	Resume screening with original modality 10 years after the false-positive screening test result.	Modeling study for the USPSTF 2021 CRC screening guideline update (26)
Surveillance	Surveillance ends at age 85 years, and intervals are based on 2 most recent colonoscopy finding.	Modeling study for the USPSTF 2021 CRC screening guideline update (26)
Survival		
Survival by stage	Cause-specific survival from SEER 2000-2003 was used, and a 7% reduction in hazard was applied (based in SEER 2010-2019) to reflect the advances in treatment.	SEER (1)

a Adapted from Vahdat et al. (25). CRC = colorectal cancer; SEER = Surveillance, Epidemiology, and End Results; USPSTF = US Preventive Services Task Force.

For the present race-specific analysis, we modified 2 key inputs of CRC-AIM listed in Table 1: all-cause mortality and survival after diagnosis. Mortality from other causes was adjusted for the Black adults using US life tables as Black adults have lower life expectancy compared with White adults. Similarly, Surveillance, Epidemiology, and End Results data show that survival rates for Black adults are lower than those for White adults even for the same cancer stages; therefore, survival after diagnosis was estimated specifically for Black adults.

### Key inputs and assumptions

We used the modeling framework and settings that were used to inform the 2021 US Preventive Services Task Force CRC screening recommendations. Similarly, the screening performance characteristics were derived from the modeling work that informed the 2021 US Preventive Services Task Force guidelines (see Table 1) (26,27). We simulated a single-birth cohort of average-risk US Black adults aged 40 years with no previous screening or CRC diagnosis until death in all the experiments.

To estimate Black-White disparities in follow-up colonoscopy completion rates after an abnormal screening test result, we conducted a comprehensive literature review, which suggested the presence of racial disparities that could be attributed to factors including insurance status and comorbidities (Supplementary Table 1, available online) (9-15). However, there is some variance in the reported magnitude of these disparities, with follow-up colonoscopy differences ranging between 3% and 20%. Although our literature review identified additional studies reporting follow-up colonoscopy differences by race, we exclusively included those presenting data for average-risk Black and White adults after an abnormal noncolonoscopy screening test from a representative US population. For example, studies including Black and White adults receiving comparable care, with similar access to specialists and insurance coverage, may not adequately represent the experience of the US Black population and are excluded. Therefore, studies analyzing large databases with a diverse set of patients (insured or uninsured; urban or rural) provide more useful information. Similarly, studies reporting race-specific odds ratios of follow-up colonoscopy rates by adjusting for factors such as obesity and insurance while ignoring race-specific disparities in these factors do not provide generalizable data. Studies that considered only a nonscreening population were also excluded. The final list informing this input parameter included 8 studies as presented in Supplementary Table 1 (available online). Using the estimates from the 2 high-quality studies (both studies estimated a value of 15%) that included a large number of participants, we assumed that the follow-up colonoscopy rate for Black adults is 15% lower than that for White adults in the base case (11,15). The proportion of the White adults who completed a follow-up colonoscopy was previously estimated as 46.7% and 71.2% after an abnormal fecal immunochemical test and multitarget stool DNA test, respectively (28). Using the 15% lower follow-up colonoscopy rates for Black adults, the proportion of the Black adults who completed a follow-up colonoscopy after an abnormal fecal immunochemical test and multitarget stool DNA test was calculated accordingly (46.7%*0.85 = 39.7% for fecal immunochemical test and 71.2%*0.85 = 60.5% for multitarget stool DNA test).

To estimate Black-White disparities in endoscopists’ adenoma detection rate, we followed a similar approach and conducted a comprehensive literature review which suggested that endoscopists’ adenoma detection rate for Black adults is approximately 3%-26% lower than that for White adults (Supplementary Table 2, available online) (20-24). Studies that did not report race-specific (Black and White adults) or screening colonoscopies (eg, those that reported outcomes only for surveillance colonoscopies) were excluded. Studies using the same dataset from earlier periods were also excluded. We only included studies that reported adenoma detection rate for average-risk Black and White adults for screening colonoscopy. The final list informing this input parameter included 5 studies as presented in Supplementary Table 2 (available online). Considering the studies that included a diverse group of participants, we conservatively assumed the endoscopists’ adenoma detection rate for Black adults is 10% lower than for White adults in the base case and conducted a sensitivity analysis on this input. CRC-AIM does not use adenoma detection rate as an input. Instead, it represents the detection of adenomas using a size-specific detection function (see Table 1), corresponding to a 29% adenoma detection rate for average-risk individuals, aligned with adenoma detection rate from a recent large retrospective cohort study in the United States (24). Therefore, endoscopists’ adenoma detection rate for average-risk Black adults was estimated as 26% (ie, 29%*0.90). Although adenoma detection rate variations may influence the impact of the effectiveness of screening colonoscopy and follow-up colonoscopy, we only addressed the adenoma detection rate disparities in screening colonoscopy because of the primary focus of our data sources on racial disparities in screening colonoscopy.

### Simulated scenarios

Our simulated scenarios considered the real-world utilization of CRC screening. In 2021 NHIS data, 54.6% of Black adults received screening colonoscopy in a prior 10-year period; 5.2% received fecal immunochemical test and/or fecal occult blood test screening in the last year, and 4.9% were screened with multitarget stool DNA test in the last 3 years (29). Because screening rates vary by age, we calculated age-specific annual prevalences for colonoscopy, fecal immunochemical test, and multitarget stool DNA test based on the overall rates from 2021 NHIS data (Supplementary Table 3, available online) (29-31). To estimate the benefit of screening, we first replicated a no screening scenario and then simulated 4 screening scenarios for Black adults aged 45-75 years (see Table 2).

**Table 2.** Summary of the simulated scenarios for Black adults

No.	Scenario name	Screening modality	Proportion of Black adults who had a follow-up colonoscopy after an abnormal stool-based test result	Adenoma detection rate for screening colonoscopy for Black adults	Proportion of screened individuals
0	No screening	None	None	Not applicable	0%
1	Screening with observed follow-up colonoscopy rates and adenoma detection rate for Black adults (15% lower follow-up colonoscopy and 10% lower adenoma detection rate)	Colonoscopy every 10 years, annual fecal immunochemical test, triennial multitarget stool DNA	39.7% after fecal immunochemical test 60.5% after multitarget stool DNA	26%	Colonoscopy: 54.6% in the previous 10 years Fecal immunochemical test: 5.2% in the previous year Multitarget stool DNA: 4.9% in the previous 3 years
2	Improved follow-up colonoscopy rates for Black adults		46.7% after fecal immunochemical test 71.2% after multitarget stool DNA	26%	
3	Improved quality of screening colonoscopy for Black adults		39.7% after fecal immunochemical test 60.5% after multitarget stool DNA	29%	
4	Improved follow-up colonoscopy rates and quality of screening colonoscopy		46.7% for fecal immunochemical test 71.2% for multitarget stool DNA	29%	

#### Scenario 1: screening with observed follow-up colonoscopy rates and adenoma detection rate for Black adults

Using real-world utilization of CRC screening (Supplementary Table 3, available online), the status quo of screening for Black adults is modeled where follow-up colonoscopy rates for Black adults were assumed to be 15% lower than those for White adults (ie, follow-up colonoscopy rates after fecal immunochemical test and multitarget stool DNA test were 39.7% and 60.5%, respectively) and the endoscopists’ adenoma detection rate for Black adults was assumed to be 10% lower than that for White adults (ie, adenoma detection rate for Black adults is 26%).

#### Scenario 2: improved follow-up colonoscopy rates

The same assumptions as in scenario 1 were used except that follow-up colonoscopy rates for Black adults were the same as those for White adults (ie, follow-up colonoscopy rates after fecal immunochemical test and multitarget stool DNA test were 46.7% and 71.2%, respectively).

#### Scenario 3: improved quality of screening colonoscopy

The same assumptions as in scenario 1 were used except that the endoscopists’ adenoma detection rate for Black adults was the same as that for White adults (29%).

#### Scenario 4: improved follow-up colonoscopy rates and quality of screening colonoscopy

Scenarios 2 and 3 were simultaneously implemented where the follow-up colonoscopy rates and adenoma detection rates were equal for both Black and White adults.

In addition to these scenarios simulating Black adults, we simulated a no screening scenario and the status quo for White adults (scenario 4) to compare the outcomes for Black and White adults. Similar to the approach used for Black adults, we used the 2021 NHIS data to determine age-specific utilization of colonoscopy, fecal immunochemical test, and multitarget stool DNA test (Supplementary Table 3, available online).

### Statistical analysis

For each experiment, we calculated the lifetime incidence, mortality, and life-years gained with screening per 1000 Black and White adults aged 45 years. We reported only mean results considering the narrow 95% confidence intervals of the simulated outcomes. Because of uncertainty in the endoscopists’ adenoma detection rate for Black adults, we conducted a sensitivity analysis on the rate of reduction in adenoma detection rate for Black adults compared with White adults ranging from 0% to 30%, with a base value of 10% (Supplementary Table 4, available online). Similarly, a sensitivity analysis for the range of follow-up colonoscopy rates for Black adults compared with White adults was conducted (ranging from a 0% to 20% rate of reduction in follow-up colonoscopy rates for Black adults compared with White adults, with a base value of 15%). We also conducted a secondary analysis that assumed perfect adherence to the screening modalities (Supplementary Table 5, available online).

## Results

**Figure 2.**
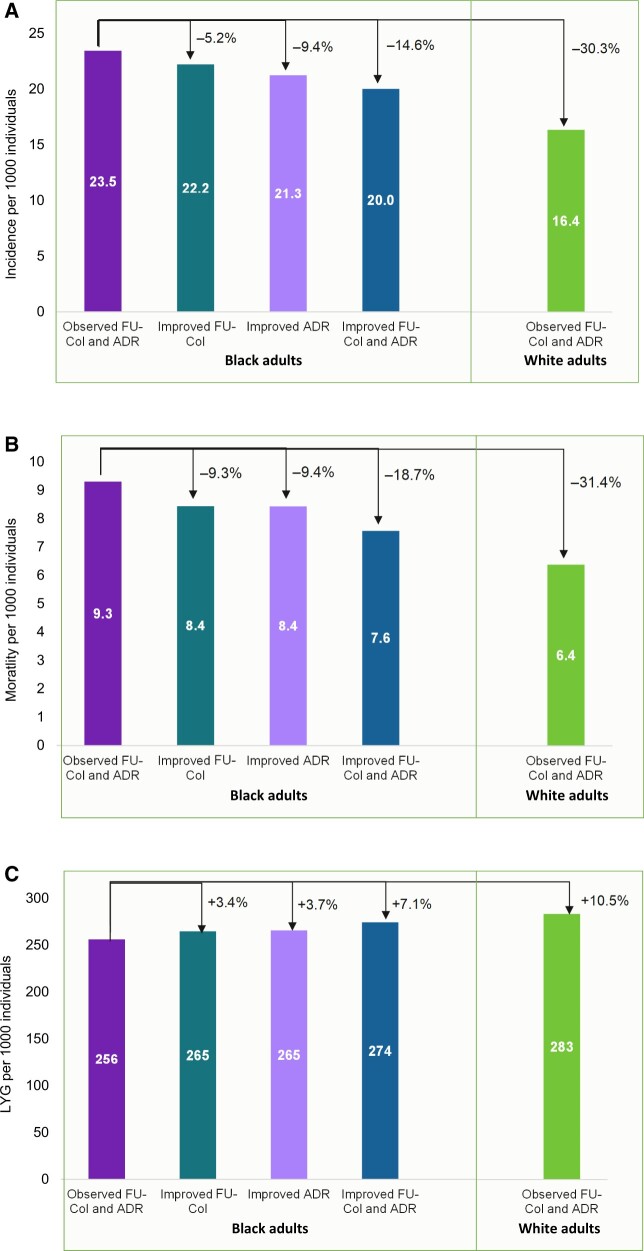
Predicted colorectal cancer outcomes per 1000 Black and White adults aged 45 years for simulated scenarios. **(A)** Incidence. **(B)** Mortality. **(C)** Life-years gained. ADR = adenoma detection rate; FU-Col = follow-up colonoscopy; LYG = life-years gained.

Lifetime CRC incidence and mortality in the absence of CRC screening were estimated as 67.5 and 34.4 per 1000 individuals among Black adults, respectively. Considering observed rates of CRC screening, with a 10% lower endoscopists’ adenoma detection rate and a 15% lower follow-up colonoscopy rate for Black adults (scenario 1), CRC incidence and mortality under CRC screening were projected as 23.5 and 9.3, respectively (Figure 2;Supplementary Table 6, available online). If Black adults had the same follow-up colonoscopy rates as White adults (scenario 2), the CRC incidence and mortality would decrease to 22.2 and 8.4, representing a 5.2% and 9.3% reduction compared with scenario 1, respectively. The elimination of racial disparities in follow-up colonoscopy rates would increase life-years gained from 256 to 265, corresponding to a 3.4% improvement. Furthermore, if Black adults had the same endoscopists’ adenoma detection rate as White adults (scenario 3), CRC incidence and mortality would reduce to 21.3 and 8.4, respectively, translating to a 9.4% reduction in both outcomes and a 3.7% improvement in life-years gained compared with scenario 1. The elimination of Black-White disparities in the follow-up colonoscopy rates and quality of colonoscopy screening (scenario 4) would result in a 14.6% reduction in CRC incidence, a 18.7% reduction in CRC mortality, and a 7.1% improvement in life-years gained. In comparison, the CRC incidence and mortality rates among White adults under CRC screening were projected as 16.4 and 6.4, respectively (Figure 2). Therefore, elimination of the Black-White disparities in endoscopists’ adenoma detection rate and follow-up colonoscopy rates would eliminate 49% (23.5-20.0 vs 16.4) and 59% (9.3-7.6 vs 6.4) of the gap between Black and White adults in CRC incidence and mortality, respectively.

**Figure 3.**
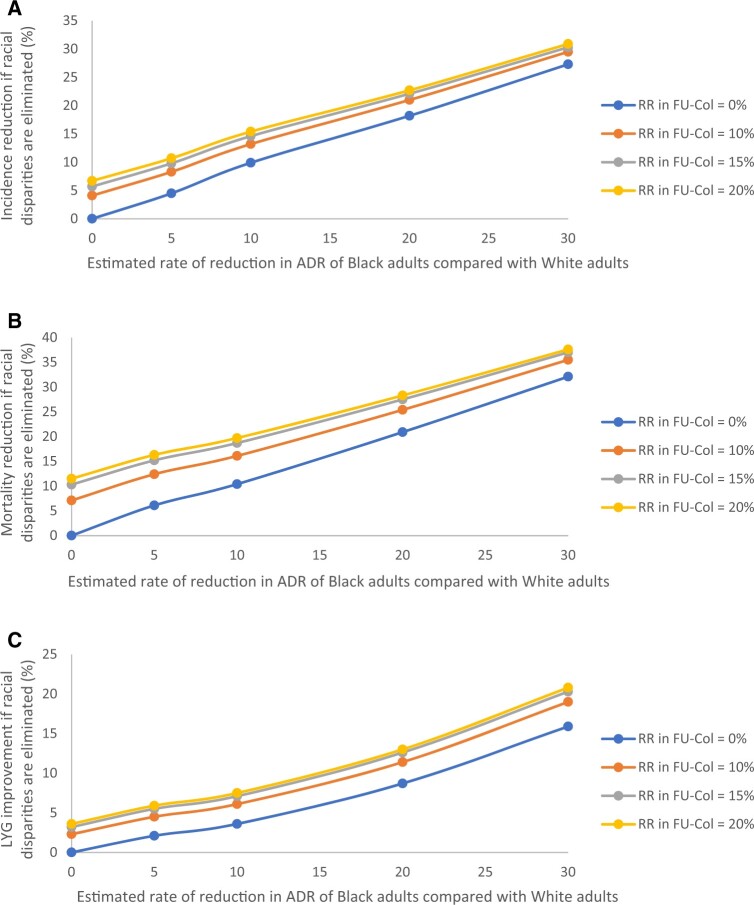
Sensitivity analysis on the impact of estimated rate of reduction in adenoma detection rate and follow-up colonoscopy rates of Black adults compared with White adults on potential improvement in colorectal cancer outcomes for Black adults if racial disparities are eliminated. **(A)** Potential reduction in incidence. **(B)** Potential reduction in mortality. **(C)** Potential improvement in life-years gained. ADR = adenoma detection rate; FU-Col = follow-up colonoscopy; LYG = life-years gained; RR = rate of reduction.

Sensitivity analysis on the rates of reduction in the follow-up colonoscopy rates and endoscopists’ adenoma detection rate for Black adults compared with White adults shows that addressing these racial disparities would reduce CRC incidence between 4% and 31%, reduce CRC mortality between 6% and 38%, and improve life-years gained because of screening between 2% and 21% (Figure 3). Secondary analysis assuming perfect adherence shows that eliminating disparities in follow-up colonoscopy and adenoma detection rate would lead to an 8%-12% reduction in CRC incidence and a 10%-13% reduction in CRC mortality for Black adults, respectively (Supplementary Table 7, available online).

## Discussion

This simulation modeling study, which considers Black-White disparities in the follow-up colonoscopy completion rates after an abnormal stool-based test and quality of colonoscopy screening, estimates that efforts focused on eliminating these gaps could improve CRC incidence, mortality, and life-years gained among Black individuals by up to 15%, 19%, and 7%, respectively. Addressing racial disparities in the follow-up colonoscopy rates would lead to a comparable level of improvement with that in the screening colonoscopy quality.

To our knowledge, no previous study has estimated the impact of improving Black-White differences in follow-up colonoscopy rates or the quality of screening colonoscopy on disparities in CRC health outcomes. Therefore, we are not able to directly compare our findings with other studies. A modeling study, which assessed the clinical and economic effects of Medicare coverage of follow-up colonoscopy after an abnormal stool-based CRC screening test with no patient cost-sharing, estimated that a 15% increase in follow-up colonoscopy rates for all patients (independent of race) would improve life-years gained by 6% (32), which is similar to our estimate of 7% increase in life-years gained for screening Black adults with fecal immunochemical test and multitarget stool DNA test. Minor differences in results are likely due to differences in the target population. The modeling study mentioned above included adults aged 65-75 years (compared with age 45-75 years in our study) and included predominantly White adults.

Our modeling analysis suggests that the elimination of the Black-White disparities in endoscopists’ adenoma detection rate and follow-up colonoscopy rates would still not raise outcomes among Black adults to those of White adults. This remaining gap may stem from Black-White differences in natural history and treatment utilization, which are not addressed in the present analysis. Furthermore, Black adults typically have higher rates of comorbidities and lower life expectancies compared with White adults.

Financial barriers and out-of-pocket costs have been identified as major factors contributing to imperfect adherence to follow-up colonoscopy (32). Recognizing the crucial role of follow-up colonoscopy rates, commercial insurers and Medicare have been mandated to eliminate out-of-pocket costs for follow-up colonoscopy as of January 2023 (32). Our study provides evidence that these initiatives have the potential to substantially reduce the racial disparities in CRC outcomes. Interventions at the system level (eg, automated referral to gastroenterologist after an abnormal stool test), provider level (eg, automated referral to colonoscopy), and patient level (eg, patient navigation) have been shown to improve follow-up colonoscopy rates, which may also be used to reduce racial disparities in follow-up colonoscopy rates (33,34).

Our findings suggest that even if Black-White disparities in the receipt of colonoscopy screening are small, disparities in outcomes can still be substantial because Black adults are more likely than White adults to receive lower-quality colonoscopy as measured by endoscopists’ adenoma detection rate. Our findings are consistent with prior studies that suggest that higher adenoma detection rate is associated with lower CRC incidence and mortality (24). Of note, colonoscopy quality is influenced by several factors, including optimal bowel preparation, cecal intubation rates, and withdrawal time, which are related to influences at the system, clinician, and patient levels. Disproportionately more Black adults receive colonoscopies from physicians with lower adenoma detection rate, reinforcing the findings from non-CRC–focused studies indicating that Black individuals are more likely to receive care from physicians in limited-resource settings and those less likely to be board certified (35). Physicians caring for Black adults have reported facing major challenges in securing access for their patients to high-quality subspecialists (35). Resolving these issues will likely require broad-reaching multifaceted interventions. Our study underscores the importance of implementing approaches to help eliminate disparities across the screening continuum and maximize the effectiveness of colonoscopy screening.

Our study has several limitations that should be considered in interpreting the results. First, the literature reported a broad range of estimates regarding the extent of racial disparities in follow-up colonoscopy rates and adenoma detection rate, and our base estimates for these inputs may not reflect all population groups or contexts. To address this limitation, we conducted an extensive sensitivity analysis using different values for these inputs to determine their impact on CRC outcomes. Nevertheless, more robust data on racial disparities in adenoma detection rate and follow-up colonoscopy rates are crucial to refine the estimates provided by this study. Secondly, although socioeconomic status and neighborhood deprivation are associated with race in terms of health-care disparities, we did not conduct a secondary analysis to address these disparities (36). This decision primarily stems from the limited availability of robust data on the impact of socioeconomic status and neighborhood deprivation on adenoma detection rate and follow-up colonoscopy rates. Therefore, this is left for future research. Thirdly, although some evidence suggests a higher risk of developing proximal adenomas for Black adults compared with White adults, we did not incorporate Black-White differences in adenoma location and CRC natural history (37). It is possible that differences in adenoma location and natural history may also contribute to racial disparities in CRC outcomes. Previous studies suggest that despite advances in metagenomic sequencing, our understanding of natural history-related racial differences is limited (38). We do not anticipate a change in our conclusions even if such differences were accounted for. In support of our results, a recent modeling study suggested that racial differences in CRC incidence do not stem from differences in biological risk for CRC (39). In addition, although 5%-30% of the CRCs are estimated to arise from serrated polyps, CRC-AIM does not represent the serrated polyp to carcinoma pathway (40). Therefore, we were unable to incorporate possible Black-White disparities in the detection of serrated polyps into the present analysis. Moreover, we only considered racial disparities in endoscopists’ adenoma detection rate for screening colonoscopy but assumed that the endoscopists’ adenoma detection rate for follow-up colonoscopy is the same between Black and White adults because of a lack of data to allow for this stratification. Finally, simulated scenarios considering real-world utilization of screening modalities assumed that individuals do not switch between screening modalities, which is unlikely to represent the real-life experience. Although we do not anticipate that this assumption would affect our conclusions, future studies should incorporate data that capture more realistic utilization of screening modalities.

In conclusion, in this simulation modeling study, we estimated that a substantial portion of the observed Black-White disparities in CRC incidence and mortality can be attributed to Black-White disparities in follow-up colonoscopy rates after an abnormal stool-based test result and quality of colonoscopy screening. Further investigation of these underappreciated contributors to racial disparities in CRC control may lead to innovative solutions for improving health equity and overall CRC outcomes in the United States.

## Supplementary Material

djae140_Supplementary_Data

## Data Availability

Additional details about the CRC-AIM simulation are available in reference (25). All data underlying this study as well as model outputs are available from the corresponding author.
