# Supplementary material for: Impact of racial disparities in follow-up and quality of colonoscopy on colorectal cancer outcomes
Source: J Natl Cancer Inst. 2024 Jul 24;116(11):1807–16. doi: 10.1093/jnci/djae140 (PMC11542987; doi:10.1093/jnci/djae140)
Supplement: djae140_Supplementary_Data [file djae140_supplementary_data.pdf]

**Supplementary Material**

# Contents:

**A. Details of the parameter estimation .......................................................................... 2**

Supplementary Table 1. Summary of literature for Black-White disparities in follow-up colonoscopy (FU-Col) completion rates after abnormal stool-based screening. ........................................................................ 2

Supplementary Table 2. Summary of literature for Black-White disparities in endoscopists' adenoma detection rate (ADR). ........................................................................ 3

Supplementary Table 3. Annual probability of age-specific utilization of different CRC screening modalities, based on NHIS 2021 data. ........................................................................ 4

Supplementary Table 4. Sensitivity of colonoscopy by size of adenoma and CRC used by CRC-AIM for different ADRs. ........................................................................ 5

**B. Additional details for the numerical results .......................................................................... 6**

Supplementary Table 5. Summary of the simulated scenarios under perfect adherence to screening for Black adults. ........................................................................ 6

Supplementary Table 6. Predicted CRC outcomes per 1,000 45-year-old Black adults for simulated scenarios. Incidence reduction, mortality reduction, and life years gained are calculated compared to the no screening scenario. ........................................................................ 7

Supplementary Table 7. Predicted CRC outcomes per 1,000 45-year-old Black adults for simulated scenarios under perfect adherence to screening. Incidence reduction, mortality reduction, and life years gained are calculated compared to the no screening scenario. ........................................................................ 8

## A. Details of the parameter estimation

**Supplementary Table 1.** Summary of literature for Black-White disparities in follow-up colonoscopy (FU-Col) completion rates after abnormal stool-based screening.

| Study (publication year) | Screening modality | Years | Number of patients* | Data source | Risk ratio |
|---|---|---|---|---|---|
| Laiyemo et al. (2010) [9] | Flexible sigmoidoscopy | 1993-2001 | 14,510 | PLCO trial | 0.88 |
| Laiyemo et al. (2015) [10] | Flexible sigmoidoscopy | 1993-2001 | 4,487 | PLCO trial | 0.90 |
| Burnett-Hartman et al. (2016) [11] | FOBT/FIT | 2010-2012 | 59,928 | PROSPR | 0.85** |
| Issaka et al. (2017) [12] | FIT | 2012-2015 | 2,238 | San Francisco Health Network | 0.87** |
| Elangovan et al. (2021) [13] | FIT | 2017-2018 | 475 | MetroHealth | 0.87** |
| Coronado et al. (2022) [14] | FIT | 2018-2020 | 1,327 | Kaiser Permanente Northwest | 0.92 |
| Mohl et al. (2023) [15] | Stool-based tests | 2017-2021 | 32,769 | Optum Labs Data Warehouse | 0.85 |
| Ciemins et al. (2024) [16] | Stool-based tests | 2018 | 20,581 | Optum Labs Data Warehouse | 0.76 |

Notes. FIT: fecal immunochemical test; FOBT: fecal occult blood test; PLCO: Prostate, Lung, Colorectal, and Ovarian Cancer Screening Trial; PROSPR: Population-based Research to Optimize the Screening Process.
Risk ratio represents the follow-up colonoscopy completion rate for Black adults compared to that for White adults. Number of patients include those with abnormal screening test results.
*When applicable, only number of Black and White individuals were included in the total number of patients with an abnormal screening test result.
**These numbers correspond to the odds ratios.

**Supplementary Table 2.** Summary of literature for Black-White disparities in endoscopists' adenoma detection rate (ADR).

| **Study (publication year)** | **Period** | **Type of colonoscopy** | **Number of patients** | **Age group** | **Data source** | **Risk ratio** |
|---|---|---|---|---|---|---|
| Diamond et al. (2011) [19] | 2005-2006 | Screening only | 17,275 | All ages | Clinical Outcomes Research Initiative database | 0.77 |
| Fedewa et al. (2017) [20] | 2002-2011 | Screening and non-screening | 61,433 | 66-75 | SEER-Medicare | 0.93 |
| David et al. (2019) [21] | 2012 | Screening only | 2,225 | 45-75 | SUNY Downstate Medical Center, New York City Health and Hospitals/Kings, and Stony Brook University Hospital | 0.74 |
| Jawitz et al. (2021) [22] | 2016-2017 | Screening only | 7,799 | All ages | Duke University Medical Center | 0.83 |
| Schottinger et al. (2022) [23] | 2011-2017 | Screening, diagnostic, and surveillance | 735,396 | 50-75 | Kaiser Permanente | 0.97* |

**Notes.** SEER: Surveillance, Epidemiology, and End Results; SUNY: State University of New York.
Risk ratio represents the ADR for Black adults compared to that for White adults.
*This number was not reported directly, instead it was calculated using the proportion of Black and White adults that are treated by physicians in different ADR categories.

**Supplementary Table 3.** Annual probability of age-specific utilization of different CRC screening modalities, based on NHIS 2021 data.

| | **Black adults** | | | **White adults** | | |
|---|---|---|---|---|---|---|
| **Age Group** | **Colonoscopy** | **FIT** | **mt-sDNA** | **Colonoscopy** | **FIT** | **mt-sDNA** |
| 45-49 | 2.74% | 3.00% | 0.17% | 2.01% | 1.67% | 0.18% |
| 50-54 | 6.28% | 3.92% | 1.19% | 5.34% | 2.94% | 1.54% |
| 55-59 | 8.03% | 7.05% | 1.51% | 8.64% | 4.72% | 2.22% |
| 60-64 | 10.95% | 6.52% | 2.49% | 11.33% | 5.28% | 2.49% |
| 65-69 | 12.15% | 7.54% | 2.74% | 12.70% | 6.08% | 2.54% |
| 70-75 | 13.18% | 7.06% | 2.48% | 12.93% | 6.28% | 2.67% |

**Supplementary Table 4.** Sensitivity of colonoscopy by size of adenoma and CRC used by CRC-AIM for different ADRs.

| | | Sensitivity of colonoscopy | | | |
|---|---|---|---|---|---|
| **Relative rate of reduction in ADR** | **ADR** | **Adenoma** | | | **CRC** |
| | | **1-5 mm** | **6-9 mm** | **10mm and over** | |
| 30% | 20% | 40% | 50% | 60% | 95% |
| 20% | 23% | 50% | 60% | 70% | 95% |
| 10% | 26% | 60% | 70% | 80% | 95% |
| 5% | 28% | 70% | 80% | 90% | 95% |
| 0% | 29% | 75% | 85% | 95% | 95% |

As noted in the main text, CRC-AIM does not use endoscopists' ADR as a direct input to represent the sensitivity of colonoscopy. Instead, sensitivity of colonoscopy is modeled as a function of adenoma size and CRC, which corresponds to an ADR value. Therefore, to implement endoscopists' ADR for Black adults, we used different sensitivity values, which are presented in Supplementary Table 4. In this table, each estimate of the relative rate of reduction in endoscopists' ADR for Black adults compared to White adults corresponds to a different ADR value. For example, the ADR corresponding to the endoscopists' ADR for White adults is 29% and our base-case estimate of 10% reduction in endoscopists' ADR for Black adults compared to White adults corresponds to an ADR of 26%.

## B. Additional details for the numerical results

In a secondary analysis, we assumed perfect adherence to CRC screening by the patients and re-ran the base-case scenarios. That is, we assumed 100% of the individuals followed USPSTF guidelines (colonoscopy every 10 years, annual FIT, triennial mt-sDNA) and re-ran Scenarios 1-3 (**Supplementary Table 5**). Because perfect adherence scenarios assumed 100% adherence to CRC screening, each simulated scenario is independently modeled and only one modality was received by all individuals for CRC screening.

**Supplementary Table 5.** Summary of the simulated scenarios under perfect adherence to screening for Black adults.

| No | Scenario name | Screening modality | Proportion of Black adults who had a FU-Col after an abnormal stool-based test result | ADR for screening colonoscopy for Black Adults | Proportion of screened individuals |
|---|---|---|---|---|---|
| **5** | Reference scenarios | | | | |
| **5a** | Annual screening with FIT using observed FU-Col rates for Black adults (15% lower FU-Col) and perfect adherence to screening | FIT | 39.7% | - | 100% annually |
| **5b** | Triennial screening with mt-sDNA using observed FU-Col rates for Black adults (15% lower FU-Col) and perfect adherence to screening | mt-sDNA | 60.5% | - | 100% triennially |
| **5c** | Screening with colonoscopy every 10 years using observed ADR for Black adults (10% lower ADR) and perfect adherence to screening | Colonoscopy | - | 26% | 100% in 10 years |
| **6** | Improved FU-Col rates for Black adults | | | | |
| **6a** | Annual screening with FIT using improved FU-Col rates for Black adults and perfect adherence to screening | FIT | 46.7% | | 100% annually |
| **6b** | Triennial screening with mt-sDNA using improved FU-Col rates for Black adults and perfect adherence to screening | mt-sDNA | 71.2% | | 100% triennially |
| **7** | Improved quality of screening colonoscopy for Black adults and perfect adherence to screening | Colonoscopy | - | 29% | 100% in 10 years |

Abbreviations: ADR: adenoma detection rate; CRC: colorectal cancer; FIT: fecal immunochemical test; FU-Col: follow-up colonoscopy; mt-sDNA: multi-target stool DNA (mt-sDNA)

**Supplementary Table 6** shows the details of the experiments for the base case and is used to generate **Figure 2** in the main text.

**Supplementary Table 6.** Predicted CRC outcomes per 1,000 45-year-old Black adults for simulated scenarios. Incidence reduction, mortality reduction, and life years gained are calculated compared to the no screening scenario.

| No | Scenario name | Incidence | Incidence reduction if racial disparities are eliminated, % | Mortality | Mortality reduction if racial disparities are eliminated, % | LYG | LYG improvement if racial disparities are eliminated, % |
|---|---|---|---|---|---|---|---|
| **0** | No Screening | 67.5 | - | 34.4 | - | 0 | - |
| **1** | Screening with observed FU-Col rates and ADR for Black adults | 23.5 | - | 9.3 | - | 256 | - |
| **2** | Improved FU-Col rates for Black adults | 22.2 | 5.2 | 8.4 | 9.3 | 265 | 3.4 |
| **3** | Improved quality of screening colonoscopy for Black adults | 21.3 | 9.4 | 8.4 | 9.4 | 265 | 3.7 |
| **4** | Improved FU-Col rates and quality of screening colonoscopy for Black adults | 20.0 | 14.6 | 7.6 | 18.7 | 274 | 7.1 |

Abbreviations. ADR: adenoma detection rate; CRC: colorectal cancer; FIT: fecal immunochemical test; FU-Col: follow-up colonoscopy; LYG: Life years gained; mt-sDNA: multi-target stool DNA (mt-sDNA).

**Supplementary Table 7.** Predicted CRC outcomes per 1,000 45-year-old Black adults for simulated scenarios under perfect adherence to screening. Incidence reduction, mortality reduction, and life years gained are calculated compared to the no screening scenario.

| No | Scenario name | Incidence | Incidence reduction if racial disparities are eliminated, % | Mortality | Mortality reduction if racial disparities are eliminated, % | LYG | LYG improvement if racial disparities are eliminated, % |
|---|---|---|---|---|---|---|---|
| **0** | No Screening | 67.5 | - | 34.4 | - | 0.0 | - |
| **5** | Reference scenarios under perfect adherence to screening | | | | | | |
| **5a** | Annual screening with FIT using observed FU-Col rates for Black adults (15% lower than that for White adults) | 33.6 | - | 14.2 | - | 227.1 | - |
| **5b** | Triennial screening with mt-sDNA using observed FU-Col rates for Black adults (15% lower than that for White adults) | 29.9 | - | 12.8 | - | 238.7 | - |
| **5c** | Screening with colonoscopy every 10 years using observed ADR for Black adults (10% lower than that for White adults) | 13.6 | - | 5.5 | - | 320.3 | - |
| **6** | Improved FU-Col rates for Black adults and perfect adherence to screening | | | | | | |
| **6a** | Annual screening with FIT using improved FU-Col rates for Black adults | 30.9 | 8.1 | 12.8 | 9.7 | 242.8 | 6.9 |
| **6b** | Triennial screening with mt-sDNA using improved FU-Col rates for Black adults | 27.1 | 9.3 | 11.5 | 10.4 | 255.9 | 7.2 |
| **7** | Improved quality of screening colonoscopy for Black adults | 12.0 | 11.9 | 4.8 | 13.3 | 330.2 | 3.1 |

Abbreviations. ADR: adenoma detection rate; CRC: colorectal cancer; FIT: fecal immunochemical test; FU-Col: follow-up colonoscopy; LYG: Life years gained; mt-sDNA: multi-target stool DNA (mt-sDNA).

Assuming perfect adherence to colonoscopy screening, CRC-AIM suggests that eliminating Black-White disparities in FU-Col rates (Scenario 6) would result in a 8% and 9% reduction in CRC incidence for FIT and mt-sDNA screening, respectively, a 10% reduction in CRC mortality, and a 7% improvement in LYG for both FIT and mt-sDNA screening (**Supplementary Table 7**). Furthermore, under the perfect adherence assumption, eliminating disparities in endoscopists' ADR (Scenario 7) would lead to an 11.9% reduction in CRC incidence, a 13.3% reduction in CRC mortality, and a 3.1% improvement in LYG for Black adults (**Supplementary Table 7**).
